# Cumulative psychosocial risk and early child development: validation and use of the Childhood Psychosocial Adversity Scale in global health research

**DOI:** 10.1038/s41390-019-0431-7

**Published:** 2019-05-18

**Authors:** Anne E. Berens, Swapna Kumar, Fahmida Tofail, Sarah K. G. Jensen, Masud Alam, Rashidul Haque, Shahria H. Kakon, William A. Petri, Charles A. Nelson

**Affiliations:** 1 Division of Developmental Medicine, Boston Children’s Hospital, Boston, MA USA; 2 Department of Pediatrics, Harvard Medical School, Boston, MA USA; 3 International Centre for Diarrhoeal Disease Research Bangladesh, Dhaka, Bangladesh; 4 Division of Infectious Diseases and International Health, University of Virginia, Charlottesville, VA USA; 5 Harvard Graduate School of Education, Cambridge, MA USA

## Abstract

**Background:**

Evidence suggests that cumulative early psychosocial adversity can influence early child development (ECD). The Childhood Psychosocial Adversity Scale (CPAS) is a novel measure of cumulative risk designed for use in global ECD research. We describe its development and assess validity from its first application in Bangladesh, where it predicts cognitive development scores among young children.

**Methods:**

Items were generated from literature review and qualitatively assessed for local relevance. Two-hundred and eighty-five mother–child dyads from an urban slum of Dhaka completed the CPAS at child ages 18, 24, 48, and/or 60 months. The CPAS was assessed for internal consistency, retest reliability, and convergent, incremental, and predictive validity.

**Results:**

The CPAS includes subscales assessing child maltreatment, caregiver mental health, family conflict, domestic violence, and household/community psychosocial risks. In Bangladesh, subscales had good internal consistency (Cronbach’s *α* > 0.70). Full-scale score had good 2-week test–retest reliability (intra-class correlation coefficient = 0.89; *F*(38,38) = 8.45, *p* < 0.001). Using multivariate regression, 48-month CPAS score significantly predicted 60-month intelligence quotient, accounting for more variance than socioeconomic status or malnutrition.

**Conclusions:**

The CPAS is a novel tool assessing cumulative childhood psychosocial risk. Evidence supports validity of its use in ECD research in Bangladesh, and ongoing work is applying it in additional countries.

## Introduction

Evidence suggests that cumulative exposure to psychosocial adversity in early life—experiences including child abuse and neglect, witnessing family violence, having a parent with untreated mental illness, or other significant psychosocial stressors—can influence developmental processes across cognitive, socio-emotional, and physical domains with implications for longitudinal health and social outcomes.^1–3^ Animal models and translational human research have suggested mechanisms by which excessive early activation of neuroendocrine stress response pathways may potentiate developmental changes in key homeostatic systems promoting chronic inflammation,^3^ immune dysfunction,^4^ and broad changes in brain structure and function impacting executive functioning, stress coping, reward processing, and higher cognition^5^ (see recent review^6^). Such mechanisms are posited to mediate epidemiological links between early adversity and increased risk of diseases ranging from depression, post-traumatic stress, and substance dependency to cancers, autoimmunity, and diabetes.^1–3^ Such work can motivate efforts to redress inequities and foster child and familial resilience and healing. In high-resource countries, in particular, this scientific foundation is driving innovation in evidence-based early childhood development (ECD) interventions.^7^

Focus on ECD is growing globally, alongside efforts to address links between psychosocial risks and child outcomes.^8^ Yet to date, much of the research on adverse childhood psychosocial experiences and child development has occurred in high-income, Western country settings.^5^ Understanding the nature, extent, and consequences of psychosocial risks across settings may be important for improving child wellbeing globally. The United Nations estimates that at least 133–275 million children globally witness violence between primary caregivers each year, while 223 million children are victims of sex trafficking.^9^ A 2016 systematic review estimated that 59% of children in developing countries had been victims of physical, emotional, or sexual violence (excluding corporal punishment) in the preceding year.^10^ Meanwhile, depression represents the leading cause of disease-related disability globally per World Health Organization (WHO) estimates with implications for caregiver mental health.^11^

One barrier to understanding and addressing developmental consequences of psychosocial stressors is a shortage of validated measurement tools assessing childhood psychosocial exposures in many settings. Data from tools like the CPAS may inform the development and prioritization of ECD interventions by assessing the prevalence of specific psychosocial risks in a community and facilitating research on related developmental harms. Among existing measures, the Adverse Childhood Experiences (ACE) Questionnaire^1^ has provided a measure of cumulative psychosocial risk in a number of research contexts, and the WHO has adapted the Adverse Childhood Experiences International Questionnaire for use globally.^12^ These tools have provided key insights into associations between early adversities and later outcomes. However, as adult retrospective self-report questionnaires, the ACE measures are not designed or validated to assess current exposures among still-young children. For this reason, they are less able to inform development of early interventions that might prevent long-term associations with poor health and social outcomes from emerging or foster healing or resilience-building efforts among young children. They also do not offer fine-grained views of the developmental environment and may be more vulnerable to recall bias as retrospective tools. Other questionnaires address specific domains of risk (for instance, exposure to child abuse^13^ or domestic violence^14^), but do not assess psychosocial risks comprehensively.

We describe development of the Childhood Psychosocial Adversity Scale (CPAS), a novel questionnaire measuring cumulative child psychosocial risk designed to be locally adapted and validated for use in ECD research in low-resource country contexts. We describe its first implementation in Bangladesh, where it effectively predicts early childhood cognitive development outcomes in a sample of children in Dhaka.

## Methods

### Instrument generation

#### Conceptual model

A conceptual model of childhood psychosocial adversity was developed from existing literature (Supplemental Fig. S1 (online)), as described in our prior review.^6^ We conceptualize *childhood adversity* as negative early life experiences associated with higher population-risk of poorer development or health outcomes. A population-risk approach acknowledges that individuals exposed to similar early risks have divergent outcomes, explored in literature on “differential susceptibility” to developmental adversity^15^ and implying that an individual’s fate is not sealed by early experiences. *Psychosocial* adversity relates specifically to child psychological processes (perceptions of threat, deprivation, fear, etc.) in interaction with the social environment, including relationships with caregivers, family, and the community.^16^ Exposures of interest identified in the literature included child abuse,^17^ psychosocial deprivation or neglect,^18^ emotionally unresponsive caregiving,^19^ caregiver depression and social isolation,^20, 21^ family violence,^22^ and other psychosocial stressors related to household poverty or community factors.^23^ Given the complexity of human social environments, our list inherently cannot capture all forms of adversity impacting children, and other groups undertaking a similar task may model risks differently.^24^ Yet, we aim to capture an adequately broad and common range of exposures to support a tool that is predictive of outcomes and useful across populations.

Our approach is situated within a framework emphasizing the importance of cumulative psychosocial risk. Within this framework, distinct ACEs may be related via shared tendency to activate neuroendocrine stress responses (i.e., via hypothalamic-pituitary-adrenal (HPA) and autonomic axes). Chronic or repeated neuroendocrine stress activation in early life, in turn, predicts brain structural and functional changes and is posited to potentiate long-term patterns of stress dysregulation, chronic inflammation, and metabolic dysfunction, described within the allostatic load paradigm.^25^ A cumulative risk approach is supported empirically by studies showing dose-response relationships between an individual’s total ACEs and later health and social outcomes.^1–3^ An implication of a cumulative risk approach is that full-scale score may represent a conceptually meaningful variable. This score would aggregate information on multiple distinct constructs to model a child’s overall burden of psychosocial stressors. Meanwhile, structuring the measure in subscales will enable future analyses with larger sample sizes to probe the empiric basis of the above hypotheses on cumulative risk by assessing the predictive power of specific subscale scores relative to each other and to full-scale score.

Several additional theoretical considerations are worth noting. While children living in settings of poverty may be at greater risk of exposure to certain psychosocial risks (for instance, community violence), we do not conceptualize low socioeconomic status (SES) as a psychosocial adversity in itself. We recognize that poverty is a broad construct that can confer risk, in part, via non-psychosocial pathways—for instance, by increasing risk of exposure to pathogens, malnutrition, and developmental toxins such as lead or arsenic. However, we do attempt to capture psychological burdens caregivers may bear related to poverty, for instance, worries related to finances and material or food insecurity. More generally, the tool may support work aiming to characterize pathways linking poverty to poorer outcomes for children. Rather than simply pathologizing children, such insights can inform efforts to foster healing and social change. It should also be noted that psychosocial protective factors are considered important but distinct from psychosocial risk factors and would be fruitfully explored in a separate measure.

Based on the conceptual model emphasizing cumulative risk and suggesting exposures potentially relevant to child development outcomes, a list of proposed instrument subscales was reviewed by academic peers from outside our study on completeness and relevance. After finalizing domains of interest, 17 existing instruments assessing relevant exposures across a variety of settings were surveyed (Supplemental Table S1 (online)), and candidate items for adaptation were identified and discussed by the study team. None were taken unchanged. In full, 169 candidate items were adapted or generated by the study team.

### Implementation in Bangladesh

#### Sample

Participants were recruited from two longitudinal birth cohorts of mother–infant dyads in Dhaka: the Performance of Rotavirus and Oral Polio Vaccines in Developing Countries (PROVIDE) cohort^26^ and the Burden of Cryptosporidiosis (Crypto) cohort.^27^ A randomly selected subset of the PROVIDE and Crypto studies were concurrently enrolled in the Bangladesh Early Adversity Neuroimaging (BEAN) Study, a longitudinal cohort study of early child neurodevelopmental risk factors and neurodevelopmental outcomes.^28^ The PROVIDE, Crypto, and BEAN studies are based at International Centre for Diarrhoeal Disease Research, Bangladesh (icddr,b), involving collaborators at icddr,b, the University of Virginia, and Boston Children’s Hospital. Recruitment for the PROVIDE and Crypto cohorts involved door-to-door visiting of all pregnant women in Mirpur during the recruitment period (2011–2015) comprising a census of over 28,000 households.^26^ Mirpur is an urban slum area in Dhaka with largely low-SES occupants, most living below the international poverty line of US: $1.90 a day and with a large share of mothers being unable to read or write. Common occupations include making handicrafts, working in garment factories, working as day laborers, driving a rickshaw, domestic service, or owning a shop or other small business. The majority of houses are made of concrete and have electricity, with overall more robust durable infrastructure than seen in rural areas, though many roads remain unpaved.

Participants in CPAS development were recruited by inviting all subjects attending scheduled visits for parent studies (PROVIDE, Crypto, and BEAN) during the period of CPAS development (September 2015–April 2018) to complete the questionnaire (Table 2). This captured children who were 18, 24, 48, and/or 60 months old. As sessions for BEAN, Crypto, and PROVIDE studies are timed to occur at specific child ages, the cohort sub-sample used for CPAS development was selected by birth date.

#### Item adaptation and translation

The relevance and completeness of the proposed subscales and candidate items in the local context were assessed using data from qualitative work with mothers and Bangladeshi study field workers in Mirpur. Sessions included semi-structured interviews (*N* = 20, including 10 with mothers and 10 with field workers) and focus groups (*N* = 8, each with 10 participants, including 4 with mothers and 4 with field workers) in Mirpur, with methods and illustrative quotes shown in Supplemental Tables S2 and S3 (online). Qualitative sessions aimed to characterize which childhood psychosocial adversities participants considered important in their communities, and how they conceptualized those phenomena. Use of both interviews and focus groups reflected a balance of two factors. First, we suspected that reflections on some topics, particularly those related to life in the community, may be enriched by interchange of ideas between community members (focus groups). Second, we suspected that some people may feel more comfortable sharing thoughts on more sensitive topics in a private, one-on-one setting (interviews). Bangladeshi field workers were included to gain the perspective of individuals with professional understanding of child development as well as deep familiarity with the local setting. Semi-structured discussion guides probed ideas and attitudes relating to child care, child discipline, family relationships, and community challenges for parents. A subset of targeted questions encouraged participants to discuss attitudes and observations about adversities identified in the conceptual model (for instance, harsh child discipline and abuse, child neglect, caregiver depression, domestic violence, family conflicts, household economic stressors, and community crime and violence), while open-ended questions probed views on positive and negative caregiving approaches and community factors impacting children in the community. In full, it was noted that all eight of the domains of psychosocial adversity identified in the CPAS conceptual model had local manifestations discussed in qualitative sessions (Supplemental Table S3 (online)). In collaboration with local study staff, CPAS items were assessed for relevance and completeness, and adjustments were made to incorporate local examples, terminology, and concepts. Candidate CPAS items were translated into Bengali and back translated to English.

#### Pretesting

CPAS candidate items were pretested with 25 mothers using cognitive interviewing, a “think-aloud” method in which participants respond to questionnaire items while articulating reasoning processes, concerns, questions, and suggestions.^29^ Cognitive interviews led to further refinement of several items to improve acceptability, relevance, and intelligibility, and to refinement of the language used in Likert response scales to make frequency intervals more comprehensible to participants, some of whom had not received formal schooling. Staff were trained in uniform administration including assessment of rating reliability.

#### Item reduction

The 169 candidate items were piloted with 53 participants at child age 48 months, the age cohort for which study visits coincided with pilot from September to November 2016. Preliminary assessment of subscale coherence and item performance used classical test theory analyses of internal consistency (Cronbach’s *α*), item-rest correlations, and response distributions. Items with inadequate distributions (e.g., mostly at ceiling or floor) were eliminated (14 items). Items with low item-rest correlations (e.g., <0.4) then were removed iteratively (66 items) with goal of achieving total administration time under 30 min for feasibility while retaining robust subscales. Items could be retained despite somewhat lower item-rest correlations (3 items) to maintain fidelity to theoretical constructs as per best practice to maintain construct validity.^29^ These items were all related to child abuse and neglect and captured more severe exposures (specifically, asking how often a caregiver had “beat [child] until he/she was cut, swollen or bruised” or told the child they “wish he/she had never been born,” or how often the child had “stayed dirty or naked for the whole day”). For 15 items asking whether a child had been exposed to particular forms of discipline or care activities, questions asking respondents to specify which caregiver (e.g., mother, father, other) had engaged in each activity were condensed to ask only about “any caregiver” to simplify the scale and improve feasibility.

### Validation

The shortened CPAS was administered to generate evidence for various types of validity (Table 1), specifically testing the proposition that the CPAS represents a valid measure of cumulative childhood psychosocial risk for use in research on early childhood development among children aged 18–60 months in Bangladesh. Of note, all 48-month-old children had received the longer CPAS version prior to item reduction based on the timing of age cohort visits. Responses to items retained on the final CPAS were included in analyses to avoid losing representation of this age group. Sensitivity analyses were performed with the goal of assessing whether inclusion of 48-month-old data for retained items may alter findings, namely if responses to retained items were influenced by the context of the longer questionnaire.

**Table 1:** Dimensions of validity assessed with hypotheses, evidence sources, and statistical criteria

Validation proposition: Use of the CPAS as a research measure assessing early psychosocial adversity as a child developmental risk factor among low-SES, urban Bangladeshi children aged 18–60 months			
Validity dimension	A priori hypotheses	Sources of evidence	Statistical criteria
Construct validity Scientific soundness of measured construct	(a) Cumulative early psychosocial stress, influenced by child and caregiver experiences, shapes human development (b) The scientific construct of psychosocial adversity has locally specific and embedded manifestations in Mirpur	Literature review	Not applicable
		Expert review	
Content validity Extent to which content captures construct	(a) Item content captures major themes in the conceptual model without extraneous content (b) Factor analysis will support a subscale structure corresponding to the conceptual model	Expert review	Significant item loading (e.g., ≥0.4) on primary factors, minimal cross-loading^30^
		Cognitive pretesting	
		EFAs	
Internal consistency Content cohesion	Subscales will show good internal consistency after final item selection	Cronbach’s *α* within subscales	Cronbach’s *α* ≥ 0.7^29^
Test–retest and inter-rater reliability Stability of scores over time/raters	(a) Total scores will have acceptable test–retest reliability (b) Total scores will have acceptable inter-rater reliability, with lower reliability than for test–retest administrations due to layered variance related to rater and occasion	Retests over 2-week interval with same interviewer (test–retest) or different (inter-rater)	Average ICC ≥ 0.75 for test–retest (excellent), ≥0.60 for inter-rater (good)^31^
Convergent and discriminant validity Agreement with similar & distinctness from dissimilar measures	(a) Subscales scores will correlate with similar instruments, likely only moderately given non-identical constructs (b) Comparator instrument scores will correlate more strongly with associated CPAS subscale than with CPAS total score	Data from CPAS and comparator instruments	Pearson’s *r* with *p* < 0.05
Predictive validity Association with outcomes	Full-scale and subscale scores significantly predict future child cognitive performance, both in bivariate analyses and when controlling for other risks.	48-month CPAS and 60-month WPPSI-IV scores	Pearson’s *r* with *p* < 0.05
Incremental validity Extent of novel value	(a) The CPAS will explore new domains of psychosocial risks while taking less time to administer than related instruments (b) It will show similar or better internal consistency	Age-matched data from CPAS and comparator measures	Cronbach’s *α* greater for CPAS greater than comparator measures

*CPAS* Global Child Adversity Scale, *EFA* exploratory factor analysis, *ICC*  intraclass correlation coefficient, *SES* socioeconomic status, *WPPSI-IV* Wechsler Preschool and Primary Scale of Intelligence, 4th Ed

#### Content validity

Subscale structure was assessed using exploratory factor analysis (EFA), a statistical method for empirically identifying the conceptual structure underlying correlations between variables. This method estimates linear regression models predicting each item score based on *n* regression coefficients, or loading factors, multiplied by an individual’s score on *n* latent variables. By this means, clusters of items can be identified for which a large share of score variance is predicted by the same latent variable (for instance, caregiver depression), which can be posited to support a subscale. A subscale was considered to be supported if all included items had significant factor loadings on the same latent construct, or factor. A simple subscale structure was also sought whereby items had low factor loadings (<0.3) for all other latent constructs (low cross-loading). Confirmatory factor analysis was deferred to future analyses based on sample size limitations. Separate EFAs were run on child-focused questions (e.g., on caregiving, abuse, or neglect), caregiver-focused items (e.g., maternal depression, domestic violence), and items on exposures in the child’s household and community context (e.g., household economic stressors, community crime, and violence). Some items were dropped from the CPAS at this stage due to low factor loadings or complex cross-loading patterns to achieve cohesive subscales. Factors with eigenvalues greater than one were retained and final models run constrained to those factors. A minimum 10:1 subject-to-item ratio was targeted as a reasonable standard expected to support reliable and meaningful models based on methodologic literature.^30^

#### Internal consistency

Subscale internal consistency and item properties were assessed by calculation of Cronbach’s *α*, item means and standard deviations (SDs), and item-rest correlations within subscales.^29^ Cronbach’s *α* was not calculated for the full CPAS as existence of multiple content domains violates homogeneity assumptions; the CPAS is not intended to measure a single, internally consistent construct, but to represent an index of theoretically related exposures. Statistics were generated separately for each child age (18, 24, 48, or 60 months) for the child-focused subscales. Internal consistency in the sample also was calculated with and without the 48-month group as sensitivity analysis.

#### Test–retest and inter-rater reliability

A subset of participants completed the measure twice over a 2-week interval with the same interviewer (39 participants, assessing test–retest reliability) or different interviewers (40 participants, assessing inter-rater reliability). Average intra-class correlation coefficients (ICCs) for within-person scores over time were calculated. The 2-week gap, selected to minimize reliability inflation from response recall, was expected to introduce acceptable variability based on true changes in underlying constructs, such that strong test–retest reliability was defined as average ICC ≥ 0.75.^31^ Interviewer–interviewee rapport was hypothesized to be an important source of rater-related error variance, so it was necessary to separate administrations over occasions (vs., for instance, having two raters score a single video-taped interview). Strong inter-rater reliability was therefore defined more permissively as average ICC ≥0.60 to account for layering of variance related to rater and occasion (two sources error variance) and true score variance.

#### Convergent validity

Pearson’s product–moment correlation coefficients (*r*) were calculated comparing CPAS subscale scores to scores on available related instruments, described below. If no instrument was available with good content overlap, a measure was selected, where possible, that assessed a construct expected to correlate with the CPAS subscale based on prior literature.

#### Predictive validity

Pearson’s *r* was also calculated comparing 48-month CPAS subscale and full-scale scores to 60-month child IQ as measured by the Wechsler Preschool and Primary Scale of Intelligence, 4th Edition (WPPSI-IV, see below).^32^ CPAS full-scale score was calculated as the simple sum of mean subscale scores in order to assess cumulative adversity. Cognitive test performance was selected as dependent variable for assessment of predictive validity based on its importance to child social functioning and the availability of quantitative measurement tools previously validated in Bangladesh and shown to be sensitive to differences in the developmental environment.^33^ This is a preliminary application performed for validation purposes, and numerous other outcome variables could be fruitfully explored.

#### Incremental validity

Internal consistency (Cronbach’s *α*, standardized to 10-item length by Spearman–Brown Prophecy Formula for comparability), predictive validity, content, and administration time was compared for the CPAS and related instruments previously used in Bangladesh, described below.

### Other measures

#### Comparator psychosocial measures

During the course of PROVIDE, Crypto, and BEAN study visits, participants completed several related questionnaires for purposes of these other studies in addition to the CPAS. At 24 and 60 months, participants completed a Bangla translation of the Edinburgh Postnatal Depression Scale (EPDS) and the Multidimensional Scale of Perceived Social Support (MSPSS). The EPDS is a 10-item measure created to screen for postpartum depression based on past-week symptoms.^34^ It is now widely used across postpartum and non-postpartum samples in a variety of contexts, including Bangladesh. Validity evidence has been published supporting the Bangla translation’s use as a postpartum clinical screen in Bangladesh,^35^ and research has linked maternal EPDS scores to impaired growth and poorer nutritional status among a sample of children in Bangladesh.^36^ The MSPSS is a 12-item measure assessing perceptions of emotional, psychological, and logistical support.^37^ There is no published validation data for use in Bangladesh, though it has been used in other South Asian country populations.^38^ BEAN participants were also assessed with the Home Observation Measurement of the Environment (HOME) Inventory at child age 24 months, a home visit-based observational measure assessing cognitive stimulation and emotional responsiveness in the child’s environment, a version of which has been adapted for use in Bangladesh by Aboud^33^ with good internal consistency. Of relevance, the WHO Multi-Country Study on Women’s Health and Domestic Violence used its questionnaire in Bangladesh.^39^ Given International Review Board (IRB) concern for substantial participant burden related to adding additional questionnaires on sensitive topics, the WHO instrument was consulted in developing CPAS items but not co-administered.

#### Socioeconomic measures

Socioeconomic data were gathered using parent report at child age 0–6 months for 18- or 24-month-old participants in CPAS validation, and at child age 25–31 months for 48- and 60-month-old. Principal component analysis was used to generate an index of SES comprised of six standardized indicators: years of maternal and paternal education, household monthly income (log transformed for normality), household crowding (people per room), household assets (for simplicity, a sum total of number of major durable goods owned, including bicycle, motorcycle, phone, sewing machine, furniture items), and paternal occupation (majority of mothers stay not employed). Paternal occupations were classified on a 5-point scale based on expert input and qualitative work as follows: 1 points if unemployed or beggar, 2 for unskilled labor in the informal sector (e.g., hawker, servant, day laborer), 3 for skilled labor in the informal sector (e.g., cobbler, tailor, carpenter) or garment factory work, 4 for formal sector professional employment (e.g., nongovernmental organization employee, government employee) or medium-sized business ownership, and 5 for large business ownership (revenue US$355 per month) or advanced professional position (e.g., lawyer, doctor). The six standardized indicators had good internal consistency (Cronbach’s *α* = 0.78), and the composite index correlated significantly with child IQ at age 48 months among 253 PROVIDE participants (*r*(251) = 0.45, *p* < 0.001) suggesting predictive validity. Given the lack of socioeconomic data collected concurrently with the CPAS, stability of predicted SES over time was also assessed in 245 PROVIDE participants from whom socioeconomic variables were collected twice, once between pregnancy month 7 and birth, and once at child age 25–31 months. Pearson’s correlation product–moment correlation coefficient between participant SES scores across the two time points indicated stability (*r*(243) = 0.90, *p* < 0.001).

#### Developmental assessment

At 48 and 60 months, BEAN and PROVIDE participants completed the WPPSI-IV, a cognitive performance measure for children 30–84 months. The WPPSI was selected as a measure of child cognitive development as it has been adapted for use in Bangladesh and has been shown to be locally acceptable and sensitive to differences in environmental factors, including features of the caregiving environment.^33^ The WPPSI-IV assesses an array of cognitive domains, including receptive and expressive vocabulary, picture memory, matrix reasoning, and identification of differences and similarities.^32^ WPPSI-IV raw scores were used in analysis vs. scaled *t*-scores (mean 100, SD 15), as score distribution differences between our sample and the normative US population used for scaling otherwise may bias analyses. It should also be noted that the WPPSI itself was derived originally for use in high-income, Western populations and IQ score differences between our sample in Bangladesh and the US reference population (the sample from which the normed score distribution was derived with a mean of 100 and SD of 25) must not be interpreted as implying clear cross-population differences in underlying developmental progress. The extent to which cultural and experiential factors (i.e., opportunities to practice the specific subsets of skills tested, familiarity with materials, etc.) may influence child performance on the WPPSI in Bangladesh is not fully known.

### Application to predict child outcomes

Multivariate ordinary least squares (OLS) linear regression models were fit predicting 60-month WPPSI-IV raw IQ score from 48-month CPAS score first as a sole predictor (Model 1), then controlling for effects of stunted growth at 48 months (Model 2), and then controlling for both stunting and SES index score (Model 3). Stunting, a marker of child malnutrition defined as height-for-age more than 2 SDs below the population mean, was included as a covariate due to its known associations with both poverty and child cognitive development outcomes, and its role as a major focus of many global health efforts aiming to improve child outcomes, including in ECD.^40^ An additional model (Model 4) included the six socioeconomic indicators individually for sensitivity analysis. Models tested for linear relationships between predictors and 60-month IQ against null hypotheses of no linear relationships. OLS assumptions of linearity, residual normality, and heteroscedasticity were assessed. These models were built as a preliminary application of the model to predict child developmental outcomes, with suggestions of future directions provided in the Discussion.

### Ethics

#### IRB approval

All activities were conducted in secure icddr,b study clinic rooms following informed consent with ethical approval from Boston Children’s Hospital and icddr,b.

#### Follow-up

Participants described psychosocial exposures (e.g., domestic violence) posing risk of serious harm in setting of limited service access, including relatively limited formal child protective services. Follow-up was designed in collaboration with Bangladeshi Principal Investigators and consultants experienced with research and services related to family violence. High-risk items were identified and those endorsing them were to be offered: (a) further assessment of needs by a clinical psychologist with expertise in family violence and related concerns, optional except in cases of concern for child maltreatment; (b) provision of confidential information about outside services (e.g., NGO, government); (c) free counseling at the icddr,b clinic from a clinical psychologist; (d) legal intervention for child protection in extreme cases, though this did not occur.

## Results

### Sample

Participants completing the CPAS (*N* = 285; Table 2) had a mean of 4.3 years of formal education (SD = 3.81), with 32% of the sample (*N* = 91) having had no formal schooling. Eighty-four percent lived below the international poverty line (US$1.90/day), with a mean income of US$1.27 per household member daily (range US$0.33–4.93, SD = US$0.68). Most mothers (78%) identified their occupation as “housewife,” while 15% worked in the home and 7% worked outside the home, most commonly in a garment factory (*N* = 6) or domestic service (*N* = 4). The most common paternal jobs were private or NGO-sector jobs (16%), owning a medium-scale business (US$118–355 monthly revenue; 15%), or making handicrafts at home (13%). As the sample was a birth cohort, all participants were biological mothers. Children included 114 identified by parents as girls and 108 as boys (51% girls).

**Table 2:** Summary of participants completing the CPAS

Child age	*N*	Cohort(s)	CPAS version	Internal consistency	Test–retest and inter-rater reliability	Convergent and discriminant validity	Predictive validity	Multivariate modeling
18 months	71	Crypto; 8 co-enrolled in BEAN	Shortened	X	X		X	
24 months	106	Crypto; all co-enrolled in BEAN	Shortened	X	X	X	X	
48 months	53	PROVIDE; 52 co-enrolled in BEAN	Long (pilot)	X	X		X	X
60 months	91^a^	PROVIDE, 52 co-enrolled in BEAN	Shortened	X	X	X	X	
Total unique	285							

*CPAS* Childhood Psychosocial Adversity Scale, *Crypto* burden of cryptosporidiosis, *BEAN* Bangladesh Early Adversity Neuroimaging Study, *PROVIDE* Performance of Rotavirus and Oral Polio Vaccines in Developing Countries ^a^Thirty-six participants completing CPAS at child age 60 months had also completed it at 48 months. For these, 60-month data were excluded from analyses when data were pooled across age groups to avoid duplicate observations per participant

### Validation analyses

#### Subscale content validity

EFA on child-focused items (18 items, subject-to-item ratio 15.83-to-1) yielded 3 factors with eigenvalues above 1 after removal of 8 items based on low factor loadings or complex cross-loadings. A final three-factor model supported subscales measuring Harsh discipline and abuse (9 items, factor loadings 0.35–0.86), Neglect (3 items, factor loadings 0.49–0.91), and Caregiver emotional unavailability (5 items, loadings 0.58–0.90). Final EFA on caregiver-focused items (25 items, subject-to-item ratio 11.40-to-1) included 4 factors with eigenvalues >1, supporting subscales measuring Depression (8 items, loadings 0.61–0.90), Social isolation (5 items, loadings 0.54–0.92), Physical intimate partner violence (7 items, loadings 0.45–0.81), and Verbal abuse and family conflict (5 items, loadings 0.61–0.87). Nineteen caregiver-focused items were removed prior to final EFA as per above. Finally, EFA on items related to the household economic and community environments supported subscales assessing Household economic stress (6 items, loadings 0.35–0.91) and Community adversity (5 items, loadings 0.45–0.70). Items and factor loadings appear in Supplemental Table S4 (online).

#### Internal consistency

Cronbach’s *α* was >0.70 for all subscales in the full sample (*N* = 285; Table 3). When the fifty-three 48 month olds (*N* = 53) who received the lengthier pilot version of the questionnaire were excluded for sensitivity analysis, Cronbach’s *α* (unitless) remained >0.7 for all subscales, and within 0.02 for all with no impact on conclusions about the strength of internal consistency. When calculated separately within age groups, Cronbach’s *α* for child-focused subscales was >0.70, with the exception of the Neglect subscale in the 24-month (Cronbach’s *α* = 0.63) and 48-month (Cronbach’s *α* = 0.68) age groups. Comparing internal consistency of CPAS subscales to existing measures, Spearman–Brown-adjusted Cronbach’s *α* (adjusted to length of 10 items for comparability) was 0.81 for the EPDS vs. 0.96 for the CPAS Depression subscale, and 0.84 for the MSPSS vs. 0.97 for the CPAS Social isolation subscale. Forty-eight-month data were not available for either the EPDS or the MSPSS to assess correlations with 60-month IQ.

**Table 3:** Classical test theory statistics, convergent validity, and predictive validity

Subscale	Items	Classical test theory statistics (*N* = 285)			Correlation with comparator instruments			Correlation with future WPPSI raw IQ^b^ (*N* = 52)
		Cronbach’s *α*	Item-rest correlations	Mean (SD) item score, 0–4 scale	Comparator instrument	*N*	Correlation coefficient (*r)*	Correlation coefficient (*r*)
Child-focused								
Harsh discipline and abuse	10	0.85	0.23–0.82	0.73 (0.69)	HOME-A^a^	106	−0.35^†^	−0.29*
Neglect	3	0.71	0.39–0.63	0.43 (0.60)	HOME-O^a^	106	−0.27**	−0.34*
Caregiver emotional unavailability	5	0.88	0.54–0.83	1.81 (1.08)	HOME-E^a^	106	−0.03	−0.43**
Maternal-focused								
Depression	8	0.95	0.75–0.84	0.99 (0.90)	EPDS	185	0.41^†^	−0.43**
Social isolation	5	0.95	0.77–0.91	0.91 (1.09)	MSPSS	185	−0.30^†^	−0.30*
Physical intimate partner violence	7	0.91	0.63–0.85	0.28 (0.52)	EPDS	185	0.20^†^	−0.47^†^
Verbal abuse and family conflict	5	0.90	0.67–0.86	1.29 (0.89)	EPDS	185	0.23^†^	−0.51^†^
Environment-focused								
Household economic stress	6	0.83	0.42–0.79	0.58 (0.68)	SES	285	−0.28^†^	−0.46^†^
Community adversity	5	0.75	0.41–0.60	0.51 (0.67)	SES	285	−0.17**	−0.30*
Full scale	**54**			7.78 (4.59)				−**0.61**^†^

*HOME* Home Observation for Measurement of the Environment Inventory, *HOME-A* HOME Avoidance of Punishment and Restriction subscale, *HOME-O* HOME Organization of the Environment subscale, *HOME-E* HOME Emotional and Verbal Responsiveness subscale, *EPDS* Edinburgh Postnatal Depression Scale, *MSPSS* Multidimensional Scale of Perceived Social Support, *WPPSI* Wechsler Preschool and Primary Scale of Intelligence 4th Edition, *IQ* Intelligence quotient

*p < 0.05, **p < 0.01, ^†^*p* < 0.001

^a^HOME and CPAS administered at child age 24 months

^b^CPAS administered at 48 months and WPPSI at 60 months to consider predictive validity

#### Convergent and discriminant validity

Of 161 participants who completed the CPAS at child age 24 and 60 months, 12 were missing MPSSS and EPDS data, all in the 60-month group (*N* = 55). Independent sample *t* tests suggested that those with missing (*N* = 12) vs. non-missing (*N* = 43) EPDS and MSPSS scores in the 60-month group did not have significantly different mean CPAS Depression subscale score (mean difference = 1.57, SE = 2.27; *t*(53) = 0.69, *p* = 0.49), Social isolation subscale score (mean difference = –0.73, SE = 1.86, *t*(53) = –0.39, *p* = 0.70), or CPAS full-scale score (mean difference = –1.10, SE = 1.59, *t*(53) = –0.69, *p* = 0.49); these observations were deleted list-wise. In remaining observations, EPDS score correlated positively and significantly with CPAS Depression score (*r*(193) = 0.43, *p* < 0.001), and with CPAS full-scale score (*r*(193) = 0.43, *p* < 0.001). MSPSS score correlated significantly with CPAS Social isolation score (*r*(193) = –0.30, *p* < 0.001) and with CPAS full-scale score (*r*(193) = 0.22, *p* = 0.002), but not with Depression score (*r*(193) = –0.13, *p* = 0.06), as predicted per convergent and discriminant validity hypotheses.

Domestic violence was expected to correlate positively with maternal depression from prior literature in Bangladesh.^14^ EPDS score correlated significantly with Physical intimate partner violence (*r*(193) = 0.29, *p* < 0.001) and Verbal abuse and family conflict (*r*(193) = 0.29, *p* < 0.001) sum scores, though with lesser magnitude than with Depression score. The HOME Avoidance of Punishment and Restriction subscale, which asked about harsh parental discipline (though does not specifically aim to assess for abuse), correlated significantly and negatively with CPAS Harsh discipline and abuse sum score (*r*(104) = –0.35, *p* < 0.001), while the HOME Organization of the Environment subscale, which considers parental attention to child activities, correlated significantly and negatively with CPAS Neglect sum score (*r*(104) = –0.27, *p* = 0.006). The HOME subscales had only partial conceptual overlap with CPAS subscales. Participant SES score correlated significantly and negatively with Household economic stress (*N* = 285, *r*(283) = –0.28, *p* < 0.001) and, more weakly, with Community adversity (*r*(283) = –0.17, *p* = 0.003; Table 3).

#### Retest reliability

In test–retest reliability assessments, average ICC for full-scale score over 2 weeks was 0.89 (95% confidence interval (CI): 0.79–0.94) in 39 retests (*N* = 10 at 18, 24, and 60 months, and *N* = 9 at 48 months) with no significant differences by child age (though small age-specific sample sizes provided low power). For inter-rater reliability, average ICC was 0.74 over 2 weeks (95% CI: 0.51–0.86) in 40 retests (*N* = 10 at 18, 24, 48, and 60 months) when including variance related to both time and person (Table 3), again with no significant differences by age. Test–retest and inter-rater ICCs at the level of subscales had wide CIs suggesting low power for these shorter scales (Supplemental Table S5 (online)).

#### Predictive validity

A single observation was deleted list-wise in models assessing predictive validity based on missing data for 60-month IQ. In 52 remaining participants, mean raw IQ was 53.04 (SD = 9.25, range 34–74). This translates to a mean scaled IQ score of 83.11 (SD = 7.95, range 67–102), where scaled scores are derived such that mean IQ is 100 and SD is 25 in the US reference population. Sixty-month raw IQ correlated significantly and negatively with 48-month CPAS full-scale score (*r*(50) = −0.61, *p* < 0.001) and all subscale scores (Table 4). Considering comparative predictive validity, scores on the EPDS and MSPSS at child age 36 months did not correlate with child IQ at age 48 months (*r*(118) = –0.01, *p* = 0.90 for EPDS; *r*(118) = 0.06, *p* = 0.50 for MSPSS) or 60 months (*r*(118) = 0.02, *p* = 0.85 for EPDS; *r*(118) = 0.04, *p* = 0.65 for MSPSS).

**Table 4:** Multivariate regression coefficients for prediction of 60-month raw IQ (*N* = 52)

	Model 1	Model 2	Model 3
Adversity score, 48 months	−1.10^†^ (0.17)	−1.09^†^ (0.19)	−0.87^†^ (0.19)
Stunted (yes/no), 48 months		−5.91* (2.39)	−5.88* (2.24)
SES composite score			1.82** (0.55)
Constant	62.47^†^ (1.80)	63.74^†^ (2.06)	62.44^†^ (1.96)
*R*^2^	0.37	0.44	0.53
DF for model *F*-statistic	1	2	3
DF for parameter *t*-statistic	50	49	48
*F*	44.35	17.30	15.00

*DF* degrees of freedom

Unstandardized coefficients (*b*) shown for each covariate with robust standard errors within parentheses

*p < 0.05, **p < 0.01, ^†^*p*  < 0.001

#### Application of the CPAS in multivariate predictive models

In multivariate models predicting 60-month child IQ from 48-month CPAS score (*N* = 53), robust standard errors were used given graphical evidence of residual heteroscedasticity over stunting. Forty-eight-month psychosocial adversity score predicted 60-month raw IQ significantly in all models. Controlling for SES composite score and child stunting (Model 3), a 1- SD increment in CPAS psychosocial adversity score predicted a 0.48 SD decrement in 60-month child IQ (*t*(48) = –4.45, *p* < 0.001), corresponding to approximately 4 scaled IQ points. This is compared to a 0.32-SD IQ increment for a 1-SD increment in SES (*b* = 1.82, SE = 0.55, *t*(48) = 3.30, *p* = 0.002; 2.5 scaled IQ points), and a 0.64-SD IQ decrement predicted by stunting (*b* *=* –5.88, SE = 2.24, *t*(48) = −2.63, *p* = 0.01; 5 scaled IQ points). Model 3 was preferred based on parsimony and clearer characterization of what appears to be a significant association between SES and child IQ (Table 4). Covariates in Model 3 accounted for 53% of variance in child IQ. A model predicting 60-month IQ from 48-month stunting and SES alone (not shown) had an *R*^2^ value of 0.33, suggesting CPAS score accounts for roughly an additional 20% of variance above the other predictors. SES accounts for roughly an additional 9% of variance over the other covariates (Model 3 vs. Model 2). Stunting accounts for roughly 8% of variance based on comparison of Model 3 to a model predicting 60-month IQ from 48-month CPAS score and SES alone (*R*^2^ = 0.45, not shown).

## Discussion

The CPAS is a comprehensive measure of child psychosocial adversity designed for implementation and validation in global health contexts, available as a free, open-source tool. In our study, it could be administered in approximately 20–30 min. Multiple sources of validity data support its use as a research tool assessing psychosocial risk factors in ECD research among young children (ages 18–60 months) in Bangladesh. In this setting, it proved to be culturally acceptable and feasible to administer during routine study visits. Psychometric analyses show good internal consistency, test–retest and inter-rater reliability, and correlations both with existing similar measures and with future child IQ. Variability in internal consistency for the Neglect subscale depending upon child age should caution against direct score magnitude comparison across age groups. Multivariate models suggest that the CPAS may serve as a useful measure assessing psychosocial risks in ECD research. Importantly, the CPAS score at child age 48 months explained a substantially greater share of variability in 60-month IQ than did child stunting status or SES. The robust psychometric properties of the CPAS are encouraging given that it was generated in a relatively efficient manner within the context of existing research activities, providing a model for how local adaptation and validation of tools could be performed in cross-cultural research. It is particularly interesting that items adapted from a variety of tools could be used in a distinct cultural context with appropriate pretesting and selection.

Considering broader implications, validated measures of childhood psychosocial adversity such as the CPAS can support assessment of the prevalence of specific types of psychosocial risk factors. These data can, in turn, aid identification of priorities for intervention to build child resilience and promote healing after early trauma. Importantly, associations observed here do not imply that early experience has a simple, deterministic effect on later outcomes. The social environment has a complex relationship to human biology, and biology, in turn, does not define an individual’s destiny. By supporting research on developmental consequences of early adversity, the CPAS may also help shed light on the full societal costs of phenomena, such as family and community violence, child maltreatment, household poverty, and poor access to mental health care. Such data may eventually foster a scientific and public consensus, and harness collective concern for children to generate political will to address important societal challenges.

Important limitations of our study and the CPAS are worth noting. Study design sought to balance generation of high-quality validity evidence with demonstration of a validation process likely to be cost-effective and feasible in future study contexts, anticipating ongoing implementation elsewhere. Incorporating CPAS development activities into existing studies made the endeavor less resource intensive, offering a test of concept for the generation and/or validation of a psychometrically rigorous tool in a relatively efficient manner. However, this design feature implies some limitations. Our samples may be characterized, to some extent, as convenience samples, though the population sampling method used in PROVIDE and Crypto Burden cohorts (inviting all pregnant women in a given geographic area in a given time frame) and birth date-based selection of CPAS participants improves representativeness. Further, we often relied on comparator measures already being administered in existing studies to avoid imposing unmanageable burden on participants. As a result, we did not administer all comparator measures at all age points and lacked data from some additional measures that might have been informative (for instance, the WHO Domestic Violence survey).

An additional limitation of our analysis to date is that sample size did not allow for several analyses of interest, including confirmatory factor analysis, assessment of differences in test–retest and inter-rater reliability at the subscale level, and by child age, in-depth assessment for differential item or subscale functioning over variables like child age or SES, or inclusion of more covariates in multivariate models. Future models with larger sample sizes may better characterize the predictive power of specific subscales relative to one another, and relative to total score. Such analyses would probe the empiric basis of the “cumulative risk” approach to modeling childhood psychosocial adversity. Another priority will be application of the CPAS to predict a wider variety of child development and clinical outcomes. Finally, we must note as a core limitation of a cohort study our inability to make causal claims from our models.

Looking ahead, efforts are now underway to implement the CPAS in additional countries with appropriate adaptation. Importantly, we note that future work may adapt CPAS items as needed for local settings based on collaborator input. Based on our experience, this may be expected to require relatively minimal change to existing items, though care is needed to ensure locally appropriate language in translations. Item wording, in particular, can then be fine-tuned through cognitive pretesting. Subsequently, a pilot version may be administered to an initial group of participants (for instance, 30–50), and any poorly performing items removed through basic classical test theory analyses such as assessment of response distributions (removing items mostly at ceiling or floor), item-rest correlations, and internal consistency. The shortened tool may then be administered to the full sample. As done here, data on retained items from pilot administrations may still be included in final analyses with appropriate discussion of assumptions in any reports of findings. As a final note, a simpler approach could also be considered in which the instrument is translated, reviewed by local collaborators for appropriateness and relevance, with perhaps small adjustments made to items, and administered, with validation analyses performed later. This approach confers greater uncertainty though could also result in an adequate tool for some purposes (e.g., if the CPAS is an adjunctive measure and resources limited). Table 5 shows a proposed framework for future implementation elsewhere.

**Table 5:** Stages of future CPAS implementation in additional contexts

Stage	Description	Minimum participants (approx.)	Contributes to final data?
Review items	Review CPAS candidate items with local collaborators and academic peers for completeness and relevance in local setting, add candidate items suggested by collaborators, and adjust existing items as needed	Not applicable	Not applicable
Pretest	Pretest candidate CPAS items with a sample of participants from the underlying population of interest using cognitive interviewing	25	No
Shorten the instrument	Pilot items with participants and use first 100 administrations for basic psychometric analyses, remove any poorly performing items	30–50	Yes
Assess validity	Administer questionnaire to participants and use first ~250 administrations to generate data to assess validity, including retest reliability if feasible; eliminate items to refine final subscales as appropriate based on EFA	250	Yes

*CPAS*  Childhood Psychosocial Adversity Scale, *EFA* exploratory factor analysis

## Conclusion

The CPAS is a novel research tool designed to measure childhood psychosocial adversities in global health research, with robust initial evidence supporting validity of its initial use in Bangladesh. Ongoing work is underway adapting it to additional contexts. It could be beneficially explored for implementation in high-resource contexts as well, including the United States, where such cumulative measures are similarly needed. This work supports efforts to bring work on early psychosocial adversity and child wellbeing more fully into the global health arena, an effort likely to be important for understanding child outcomes. It also contributes efforts to improve measurement of early psychosocial risk in ECD research more generally.

## Supplementary information

Supplementary Figure S1

Supplementary Material
