# Supplementary figures and images for: Cumulative psychosocial risk and early child development: validation and use of the Childhood Psychosocial Adversity Scale in global health research
Source: Pediatr Res. 2019 May 18;86(6):766–75. doi: 10.1038/s41390-019-0431-7 (PMC6859196; doi:10.1038/s41390-019-0431-7)

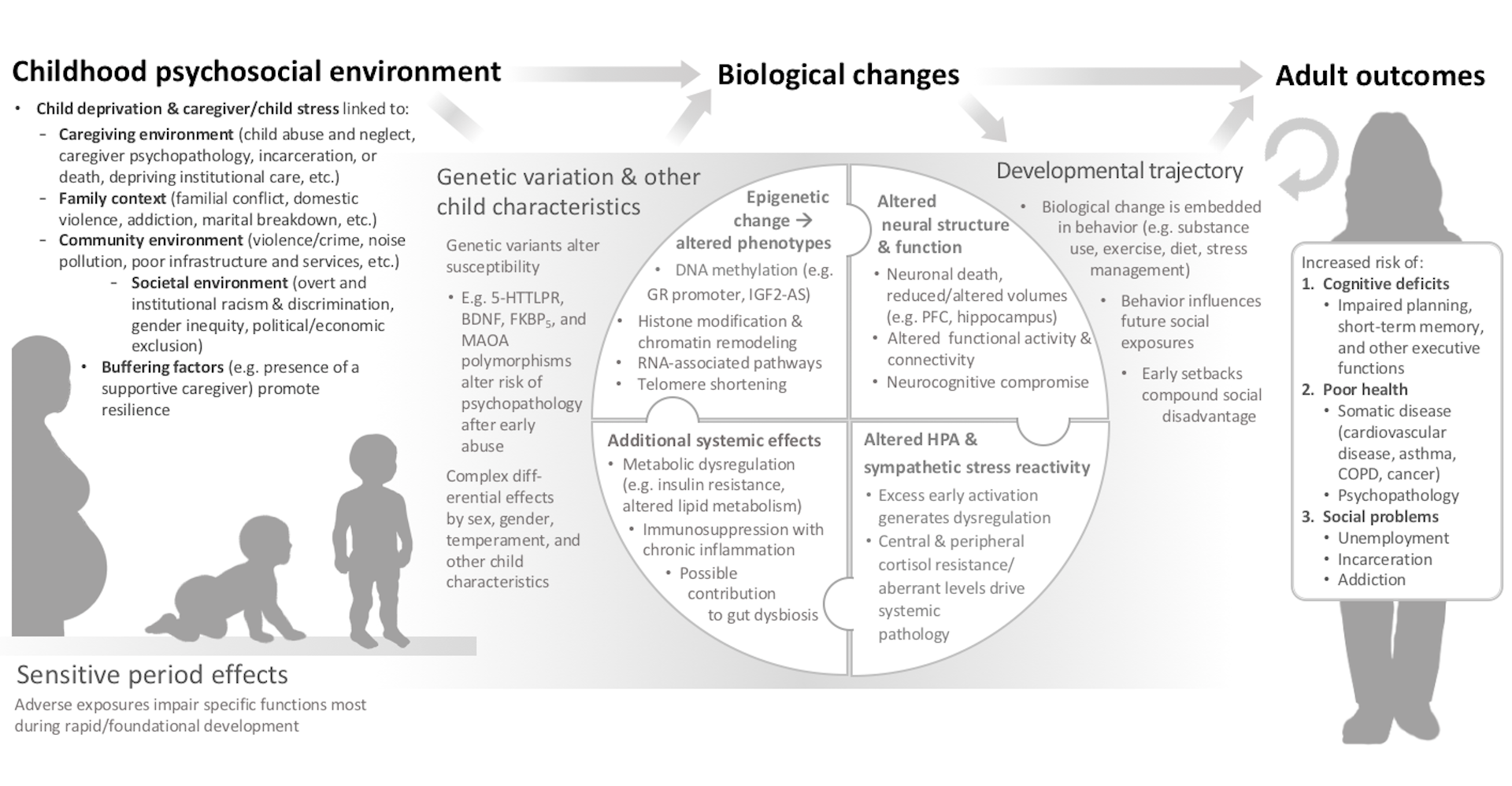

Supplement: Supplementary file 1 — Supplementary Figure S1 [file 41390_2019_431_MOESM1_ESM.png]
