# Supplementary material for: Cumulative psychosocial risk and early child development: validation and use of the Childhood Psychosocial Adversity Scale in global health research
Source: Pediatr Res. 2019 May 18;86(6):766–75. doi: 10.1038/s41390-019-0431-7 (PMC6859196; doi:10.1038/s41390-019-0431-7)
Supplement: Supplementary file 2 — Supplementary Material [file 41390_2019_431_MOESM2_ESM.docx]

**Figure S1. Conceptual model of the biological embedding of early psychosocial exposures**

See separate high-resolution figure file.

| **Table S1. Prior instruments consulted during item generation** | |
| --- | --- |
| General  Adverse Childhood Experiences Questionnaire (1)  WHO Adverse Childhood Experiences International Questionnaire (2)  Child abuse  Conflict Tactics Scale Parent-Child version (3)  International Society for Prevention of Child Abuse & Neglect Child Abuse Screening Tool, Parent Version (4)  Multiple Index Cluster Survey Household Questionnaire (5)  Child neglect & emotional unavailability  Home Observation Measurement of the Environment Inventory (6)  Multidimensional Neglectful Behavior Scale (7)  Multiple Index Cluster Survey Household Questionnaire (5)  Family Care Indicators (8) | Caregiver depression, isolation and stress  Center for Epidemiological Studies Depression Scale (9)  Edinburgh Postpartum Depression Scale (10)  Multidimensional Scale of Perceived Social Support (11)  Perceived Stress Scale (12)  Tension Scale (13)  Household exposures  Household Food Insecurity Access Scale (14)  Multiple Index Cluster Survey Household Questionnaire (5)  Intimate Partner Violence  USAID Demographic & Health Surveys Domestic Violence Module (15)  Psychological Maltreatment of Women Inventory (16)  WHO Multi-Country Domestic Violence Surveys (17) |

| **Table S2. Methods used in informative qualitative work** |
| --- |
| Sessions were conducted by study staff in Bangla using interview guides with prompts probing local norms, attitudes, and practices relating to caregiving and to child and caregiver experiences of stress. Sessions were audio-recorded, transcribed, translated, and analyzed using NVivo 11 (QSR International Pty Ltd, 2015). A qualitative content analysis was conducted integrating inductive generation of codes with deductive application of themes drawn from literature on psychosocial stress and child development (18, 19). Codes were generated by two qualitative analysts on the team with input from Bangladeshi and US collaborators, and initial transcripts were double coded by the lead author and a Bangladeshi research assistant at Boston Children’s Hospital. Codes were refined until good inter-rater agreement was achieved (κ>0.75). Subsequently, roughly 15% of the 28 transcripts were double coded and compared for discussion between the coders. |

| **Table S3. Conceptual model of adversity domains with selected qualitative findings** | | | |
| --- | --- | --- | --- |
| **Domains of adversity** | **Examples of themes emerging in qualitative work** | **Quote illustrating one or more key themes** |  |
| **Poor caregiving quality & child neglect** | Many hesitated to identify “poor” caregiving citing economic constraints faced by parents, but did emphasize that parents had to do their best within constraints. | *“As an example of poor caregiving, there is a mother in our house who has to go to work, leaving her 3-month-old baby with her sister-in-law. So, the baby does not get enough care. For instance, the baby needs milk but doesn’t get it." -*Parent  *“If I have five taka [USD$0.06], then I have to manage everything with that five taka; and if instead of spending it on my family, I spend it on myself, then the education of my children will suffer.”* -Parent |  |
| **Harsh child discipline & abuse** | Corporal punishment was described as widespread and generally acceptable if mild. Using swear words or slang with children was considered wrong, as was more severe physical punishment causing injury. | R:*“ I saw a mother throw a chair she had been sitting on at her child. By this, the child can get hurt...If my child does wrong, I can lock her in a room, can beat her slightly with a stick…This type of discipline is okay.”* [I:*“But that which injures a child is not ok?"*] R:*“That is not ok. My child can be hurt very badly because of me, no?" -*Parent |  |
| **Marital & family conflict** | Marital disputes represented a significant source of maternal stress. Criticism of wife by in-laws and extended family was seen as a common source of distress and conflict. | *“There are some husbands and wives who fight a lot…Lately, there are cases in the community where the wife takes poison because of fights with her husband.”* - Parent  *“Often, a mother-in-law treats her daughter-in-law very badly…asking her to do every household chore, but still she’s not satisfied…so the husband starts beating his wife.” -*Parent |  |
| **Domestic violence** | Verbal and physical abuse of mothers was described as very common, sometimes causing serious injuries. | *“Husbands beat their wives with whatever they have in front of them. My child’s father beats me in that way…If he gets a floor cleaner or stick, he beats me with that.” -*Parent |  |
| **Maternal social support** | A number of participants described feeling isolated, devalued, and unseen. | *"[In families] it’s like we do not have any value. No one listens to us and our words are not valued too." –* Staff |  |
| **Household economic stressors** | Participants described household poverty as a major stressor and determinant of the quality of caregiving that parents are able to provide to children. | "*While parents are in the house there is no electricity. So they are busy arranging straw for fuel and cooking. So, we usually found them too busy to play with children.”*-Staff |  |
| **Maternal depression** | Participants readily launched into discussion of mental health problems in the community, though they did not generally use clinical terms or concepts. | *“At times I feel very sad, very low; I cannot sleep at night, I cannot eat...I don’t even like my husband. I don’t even like my children, I feel like going away, I feel very bad, my body becomes weak…”* [I: *“So what do you call this?”*] *“To us, it is called sorrow. It is our sadness, misery.”-*Parent |  |
| **Community adversities** | Participants described stressors in the community, including theft, gang activity, drug trading, unsafe roads, political violence, and more rarely, cases of physical and sexual assault. | *“What will happen to him when he goes out on the street? Will he be hit by a car? Will he get into trouble? Will he be mugged by someone? Has anyone snatched his valuables, mobile phone, money?”*-Parent |  |

| **Table S4. Items & factor loadings from exploratory factor analyses** | | | | | | | | | | | | | | |  |
| --- | --- | --- | --- | --- | --- | --- | --- | --- | --- | --- | --- | --- | --- | --- | --- |
|  |  | **Child-Focused Items** | | |  |  | **Caregiver-Focused Items** | | | |  |  | **Household/community-Focused Items** | | |
| **Item** | **Question (paraphrased)** | **Factor1**  Child abuse | **Factor2**  Low warmth | **Factor3**  Child neglect |  |  | **Factor1** Depression | **Factor2** Social isolation | **Factor3** Physical IPV | **Factor4** Family conflict |  |  | **Factor2**  Economic  stress | **Factor2**  Community  adversities | |
| *How often in the past 1 month did any of  [child]’s caregivers do the following with [child]…* | |  |  |  |  |  |  |  |  |  |  |  |  |  | |
| 16 | Threatened to beat | 0.85 |  |  |  |  |  |  |  |  |  |  |  |  | |
| 9 | Beat lightly | 0.85 |  |  |  |  |  |  |  |  |  |  |  |  | |
| 13 | Criticized with names | 0.68 |  |  |  |  |  |  |  |  |  |  |  |  | |
| 17 | Frightened for punishment | 0.68 |  |  |  |  |  |  |  |  |  |  |  |  | |
| 14 | Cursed with slang | 0.64 |  |  |  |  |  |  |  |  |  |  |  |  | |
| 15 | Shook | 0.60 |  |  |  |  |  |  |  |  |  |  |  |  | |
| 10 | Beat harshly | 0.54 |  |  |  |  |  |  |  |  |  |  |  |  | |
| 11 | Beat with object | 0.48 |  |  |  |  |  |  |  |  |  |  |  |  | |
| 12 | Beat so cut, swollen, or bruised | 0.34 |  |  |  |  |  |  |  |  |  |  |  |  | |
| 18 | Expressed wish that he/she would die | 0.34 |  |  |  |  |  |  |  |  |  |  |  |  | |
| *How often in the past 1 month did any of  [child]’s caregivers do the following with [child]…* | |  |  |  |  |  |  |  |  |  |  |  |  |  |  |
| 5 | Called him/her sweet names (-) |  | 0.90 |  |  |  |  |  |  |  |  |  |  |  |  |
| 6 | Gave praise (-) |  | 0.82 |  |  |  |  |  |  |  |  |  |  |  |  |
| 7 | Showed physical affection (-) |  | 0.82 |  |  |  |  |  |  |  |  |  |  |  |  |
| 4 | Talked or chatted for enjoyment (-) |  | 0.74 |  |  |  |  |  |  |  |  |  |  |  |  |
| 8 | Took on lap or bosom to quiet (-) |  | 0.58 |  |  |  |  |  |  |  |  |  |  |  |  |
| *How often in the past 1 month was [child]…* | |  |  |  |  |  |  |  |  |  |  |  |  |  |  |
| 2 | Left in house >1hr without supervision |  |  | 0.91 |  |  |  |  |  |  |  |  |  |  |  |
| 1 | Left in care of other child >1hr |  |  | 0.90 |  |  |  |  |  |  |  |  |  |  |  |
| 3 | Left free outside |  |  | 0.48 |  |  |  |  |  |  |  |  |  |  |  |
| *In the past 6 months, how often did problems in  your mind cause you to feel…* | |  |  |  |  |  |  |  |  |  |  |  |  |  |  |
| 40 | Like you don't like anything |  |  |  |  |  | 0.90 |  |  |  |  |  |  |  |  |
| 39 | In no mood to talk or socialize |  |  |  |  |  | 0.88 |  |  |  |  |  |  |  |  |
| 42 | Unable to care for or pay attention to [child] |  |  |  |  |  | 0.78 |  |  |  |  |  |  |  |  |
| 43 | Unable to feel joy with [child] |  |  |  |  |  | 0.72 |  |  |  |  |  |  |  |  |
| 41 | Tearful or crying a lot |  |  |  |  |  | 0.72 |  |  |  |  |  |  |  |  |
| 38 | Physical difficulties |  |  |  |  |  | 0.64 |  |  |  |  |  |  |  |  |
| 37 | Anxious or worried |  |  |  |  |  | 0.61 |  |  |  |  |  |  |  |  |
| 36 | Persistent sadness or sorrow |  |  |  |  |  | 0.59 |  |  |  |  |  |  |  |  |
| Factor loadings <0.30 not shown; cross-loading of item onto non-primary factor italicized; (-) indicates item reverse coded; IPV=intimate partner violence | | | | | | | | | | | | | | |  |

| **Table S4 (Continued). Items & factor loadings from exploratory factor analyses** | | | | | | | | | | | | | | |  |
| --- | --- | --- | --- | --- | --- | --- | --- | --- | --- | --- | --- | --- | --- | --- | --- |
|  |  | **Child-Focused Items** | | |  |  | **Caregiver-Focused Items** | | | |  |  | **Household/community-Focused Items** | | |
| **Item** | **Question (paraphrased)** | **Factor1**  Child abuse | **Factor2**  Low warmth | **Factor3**  Child neglect |  |  | **Factor1** Depression | **Factor2** Social isolation | **Factor3** Physical IPV | **Factor4** Family conflict |  |  | **Factor2**  Economic  stress | **Factor2**  Community  adversities | |
| *How often in the past 6 months did you feel that…* | |  |  |  |  |  |  |  |  |  |  |  |  |  | |
| 28 | You had no one to show you kindness |  |  |  |  |  |  | 0.92 |  |  |  |  |  |  | |
| 26 | You had no one to cheer you up |  |  |  |  |  |  | 0.90 |  |  |  |  |  |  | |
| 27 | You had no one to give advice |  |  |  |  |  |  | 0.89 |  |  |  |  |  |  | |
| 25 | You were all alone |  |  |  |  |  |  | 0.82 |  |  |  |  |  |  | |
| 24 | Home was hell or you had no peace at home |  |  |  |  |  |  | 0.54 |  |  |  |  |  |  | |
| *How often in the past 6 months did it happen that your partner…* | |  |  |  |  |  |  |  |  |  |  |  |  |  |  |
| 21 | Insulted you |  |  |  |  |  |  |  | 0.83 |  |  |  |  |  |  |
| 23 | Yelled at you when [child] could see or hear |  |  |  |  |  |  |  | 0.82 |  |  |  |  |  |  |
| 22 | Made you feel ashamed/humiliated |  |  |  |  |  |  |  | 0.76 |  |  |  |  |  |  |
| 20 | Quarreled with you a lot |  |  |  |  |  |  |  | 0.69 |  |  |  |  |  |  |
| 19 | Was very critical of you |  |  |  |  |  |  |  | 0.57 |  |  |  |  |  |  |
| 30 | Beat you with an object |  |  |  |  |  |  |  |  | 0.81 |  |  |  |  |  |
| 32 | Made you feel afraid for your life |  |  |  |  |  |  |  |  | 0.79 |  |  |  |  |  |
| 34 | Threatened to beat you |  |  |  |  |  |  |  |  | 0.78 |  |  |  |  |  |
| 33 | Kicked you, dragged you, or beat you up |  |  |  |  |  |  |  |  | 0.78 |  |  |  |  |  |
| 31 | Beat you harshly |  |  |  |  |  |  |  |  | 0.77 |  |  |  |  |  |
| 29 | Beat you a little |  |  |  |  |  |  |  |  | 0.72 |  |  |  |  |  |
| 35 | Beat you when [child] could see or hear |  |  |  |  |  |  |  | *0.37* | 0.45 |  |  |  |  |  |
| *How often in the past 6 months did you feel tension or worry because/because of…* | |  |  |  |  |  |  |  |  |  |  |  |  |  |  |
| 47 | You could not buy [child] nutritious food |  |  |  |  |  |  |  |  |  |  |  | 0.91 |  |  |
| 48 | You could not buy [child] needed basics |  |  |  |  |  |  |  |  |  |  |  | 0.85 |  |  |
| 45 | You could not afford enough food |  |  |  |  |  |  |  |  |  |  |  | 0.81 |  |  |
| 46 | You went hungry because of lack of money |  |  |  |  |  |  |  |  |  |  |  | 0.63 |  |  |
| 44 | You did not have enough money |  |  |  |  |  |  |  |  |  |  |  | 0.54 |  |  |
| 49 | Class-based discrimination |  |  |  |  |  |  |  |  |  |  |  | 0.35 |  |  |
| 50 | Violence in the community |  |  |  |  |  |  |  |  |  |  |  |  | 0.70 |  |
| 53 | Gangs or drug trading in the community |  |  |  |  |  |  |  |  |  |  |  |  | 0.69 |  |
| 51 | Crime in the community |  |  |  |  |  |  |  |  |  |  |  |  | 0.68 |  |
| 54 | Eve teasing in the community |  |  |  |  |  |  |  |  |  |  |  |  | 0.60 |  |
| 52 | Trash or poor sanitation in the community |  |  |  |  |  |  |  |  |  |  |  |  | 0.45 |  |
| Factor loadings <0.30 not shown; cross-loading of item onto non-primary factor italicized; (-) indicates item reverse coded; IPV=intimate partner violence | | | | | | | | | | | | | | |  |

| **Table S5. Subscale test-retest and inter-rater reliability** | | | | | | | | | | | | | | | |
| --- | --- | --- | --- | --- | --- | --- | --- | --- | --- | --- | --- | --- | --- | --- | --- |
|  |  | Test-retest reliability  (N=39) | | | | |  | | | Inter-rater reliability  (N=40) | | | | |  |
|  |  | ICC | | 95% CI | |  | | | ICC | | | 95% CI | |  |  |
| ***Child-focused*** |  |  | |  | |  | | |  | | |  | |  |  |
| Harsh discipline & abuse |  | 0.88^†^ | | 0.77-0.94 | |  | | | 0.77^†^ | | | 0.57-0.88 | |  |  |
| Neglect |  | 0.74^†^ | | 0.50-0.86 | |  | | | 0.72^†^ | | | 0.46-0.85 | |  |  |
| Caregiver emotional unavailability |  | 0.81^†^ | | 0.63-0.90 | |  | | | 0.75^†^ | | | 0.53-0.87 | |  |  |
| ***Caregiver-focused*** |  |  | |  | |  | | |  | | |  | |  |  |
| Depression |  | 0.75^†^ | | 0.52-0.87 | |  | | | 0.50* | | | 0.05-0.74 | |  |  |
| Social isolation |  | 0.62*** | | 0.28-0.80 | |  | | | 0.50* | | | 0.06-0.74 | |  |  |
| Physical intimate partner violence |  | | 0.83^†^ | | 0.67-0.91 | | |  | | | 0.66^†^ | | 0.36-0.82 | |  |
| Family conflict |  | 0.81^†^ | | 0.64-0.90 | |  | | | 0.67^†^ | | | 0.38-0.83 | |  |  |
| ***Environment-focused*** |  |  | |  | |  | | |  | | |  | |  |  |
| Household economic stress |  | 0.90^†^ | | 0.81-0.95 | |  | | | 0.79^†^ | | | 0.61-0.89 | |  |  |
| Community adversity |  | 0.70^†^ | | 0.42-0.84 | |  | | | 0.49* | | | 0.04-0.73 | |  |  |
| **Full scale** |  | 0.89^†^ | | 0.79-0.94 | |  | | | 0.74^†^ | | | 0.40-0.86 | |  |  |
| *p<0.05 **p<0.01 ^†^p<0.001  ICC=Average intraclass correlation coefficient; CI=Confidence interval | | | | | | | | | | | | | | | |

**References for supplementary materials:**

1. Felitti VJ, Anda RF, Nordenberg D, et al. Relationship of childhood abuse and household dysfunction to many of the leading causes of death in adults. The Adverse Childhood Experiences (ACE) Study. Am J Prev Med 1998;14(4):245-58.
2. World Health Organization. Adverse Childhood Experiences International Questionnaire. Geneva: World Health Organization, 2018.
3. Straus MA, Hamby SL, Finkelhor D, et al. Identification of child maltreatment with the Parent-Child Conflict Tactics Scales: development and psychometric data for a national sample of American parents. Child Abuse Negl 1998;22(4):249-70.
4. Runyan DK, Dunne MP, Zolotor AJ, et al. The development and piloting of the ISPCAN Child Abuse Screening Tool-Parent version. Child Abuse Negl 2009;33(11):826-32.
5. UNICEF. Multiple Index Cluster Surveys 5 (MICS5): Household Questionnaire. New York: UNICEF, 2013.
6. Caldwell B, Bradley R. Home Observation for Measurement of the Environment (HOME)-revised edition. Little Rock, AR: University of Arkansas, 1984.
7. Kantor GK, Holt MK, Mebert CJ, et al. Development and preliminary psychometric properties of the multidimensional neglectful behavior scale-child report. Child Maltreat 2004;9(4):409-28.
8. Hamadani JD, Tofail F, Hilaly A et al. Use of family care indicators and their relationship with child development in Bangladesh. J Health Popul Nutr 2010;28(1):23-33.
9. Radloff LS. The CES-D scale: A self-report depression scale for research in the general population. Appl Psychol Meas 1977;1(3):385-401.
10. Cox JL, Holden JM, Sagovsky R. Detection of postnatal depression: development of the 10-item Edinburgh Postnatal Depression Scale. Br J Psychiatry 1987;150(6):782-86.
11. Zimet GD, Dahlem NW, Zimet SG, Farley GK. The Multidimensional Scale of Perceived Social Support. J Pers Assess 1988;52(1):30-41.
12. Cohen S, Kamarck T, Mermelstein R. A global measure of perceived stress. J Health Soc Behav. 1983;1:385-96.
13. Swindale A, Bilinsky P. Development of a universally applicable household food insecurity measurement tool: process, current status, and outstanding issues. J Nutrition 2006;136(5):1449S–52S
14. Karasz A, Patel V, Kabita M, Shimu P. Tension in South Asian women. Prog Community Health Partnersh 2013;7(4):429-41.
15. USAID. Demographic and Health Surveys Toolkit: Domestic Violence Module. Washington, DC: United States Agency for International Development, 2008.
16. Tolman RM. The validation of the Psychological Maltreatment of Women Inventory. Violence Vict 1999;14(1):25-37.
17. Ellsberg M, Jansen HAFM, Heise L, et al. Intimate partner violence and women's physical and mental health in the WHO multi-country study on women's health and domestic violence: an observational study. Lancet 2008;371(9619):1165-72.
18. Onwuegbuzie AJ, Dickinson WB, Leech NL, Zoran AG. A qualitative framework for collecting and analyzing data in focus group research. Int J Qual Methods 2009;8(3):1-21.
19. Hsieh H-F, Shannon SE. Three approaches to qualitative content analysis. Qual Health Res 2005;15(9):1277-88.
